# Supplementary material for: Membrane Localization and Phosphorylation of Indoleamine 2,3-Dioxygenase 2 (IDO2) in A549 Human Lung Adenocarcinoma Cells: First Steps in Exploring Its Signaling Function
Source: Int J Mol Sci. 2023 Nov 12;24(22):16236. doi: 10.3390/ijms242216236 (PMC10671178; doi:10.3390/ijms242216236)
Supplement: Supplementary file 1 [file ijms-24-16236-s001.zip › ijms-2645451-supplementary.pdf]

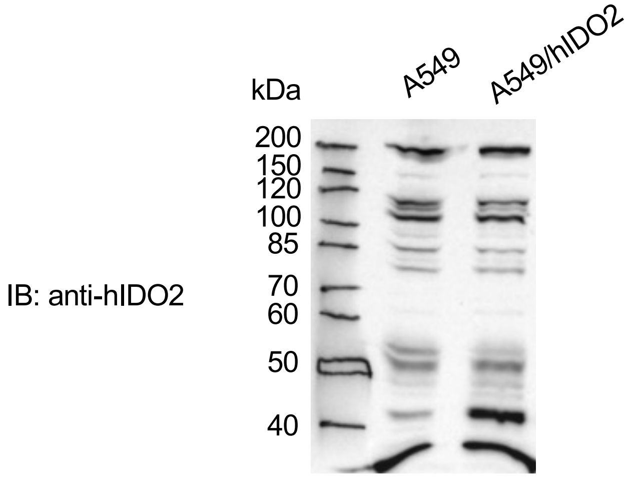

Figure S1. Full-size image of the Western blotting analysis of IDO2 protein expression (MW= 42 kDa) in A549 and A549/hIDO2 whole-cell lysates with the rabbit polyclonal anti-hIDO2 antibody I23O2.
